# Opposite Association Between Wet‐Bulb Globe Temperature With Central Obesity in Different Geographic Regions in Taiwan

**DOI:** 10.1155/ije/6443831

**Published:** 2026-02-24

**Authors:** Sheng-Hao Chang, Wei-Yu Su, Ping-Hsun Wu, Chen-Yang Hsiao, Ming-Yen Lin, Yi-Wen Chiu, Jer-Ming Chang, Chih-Da Wu, Szu-Chia Chen

**Affiliations:** ^1^ Department of Post Baccalaureate Medicine, Kaohsiung Medical University, Kaohsiung, Taiwan, kmu.edu.tw; ^2^ Division of Nephrology, Department of Internal Medicine, Kaohsiung Medical University Hospital, Kaohsiung Medical University, Kaohsiung, Taiwan, kmu.edu.tw; ^3^ Faculty of Medicine, College of Medicine, Kaohsiung Medical University, Kaohsiung, Taiwan, kmu.edu.tw; ^4^ Center for Big Data Research, Kaohsiung Medical University, Kaohsiung, Taiwan, kmu.edu.tw; ^5^ Department of Geomatics, College of Engineering, National Cheng Kung University, Tainan, Taiwan, ncku.edu.tw; ^6^ National Institute of Environmental Health Sciences, National Health Research Institutes, Miaoli, Taiwan, nih.gov; ^7^ Research Center for Precision Environmental Medicine, Kaohsiung Medical University, Kaohsiung, Taiwan, kmu.edu.tw; ^8^ Innovation and Development Center of Sustainable Agriculture, National Chung Hsing University, Taichung, Taiwan, nchu.edu.tw; ^9^ Chronic Diseases and Health Promotion Research Center, Chang Gung University of Science and Technology, Kaohsiung Medical University, Puzi, Taiwan, cgust.edu.tw; ^10^ Department of Internal Medicine, Kaohsiung Municipal Siaogang Hospital, Kaohsiung Medical University, Kaohsiung, Taiwan, kmu.edu.tw

**Keywords:** central obesity, different geographic region, Taiwan biobank, wet-bulb globe temperature

## Abstract

**Objective:**

Despite the known effects of global warming and rising ambient temperatures, the potential influence of environmental heat stress on central obesity remains underexplored, particularly in subtropical and tropical regions such as Taiwan. This study investigates the association between wet‐bulb globe temperature (WBGT) with central obesity across the main island of Taiwan.

**Methods:**

Using data from 120,424 participants in the Taiwan Biobank, central obesity was assessed via waist circumference (WC), with central obesity defined as WC ≥ 80/90 cm in women/men. WBGT exposure data were derived using high‐resolution spatial‐temporal models. The analysis examined WBGT values during two time periods: work (8:00 a.m.–5:00 p.m.) and midday (11:00 a.m.–2:00 p.m.).

**Results:**

In central Taiwan, each 1°C increase in average midday period WBGT was significantly associated with a high prevalence of central obesity (odds ratio [OR]: 1.048, *p* = 0.009), whereas each 1°C rise in both midday and work period WBGT in southern Taiwan was significantly associated with a low prevalence of central obesity (OR: 0.929, *p* < 0.001, and 0.954, *p* < 0.001, respectively). However, no significant associations were observed between central obesity with WBGT in northern or eastern Taiwan.

**Conclusions:**

There are significant regional disparities in the association between WBGT with central obesity in Taiwan, emphasizing the need for tailored public health strategies and climate‐adaptive interventions to mitigate the risk of obesity across different environments.

## 1. Introduction

Central obesity is defined as the accumulation of excess visceral fat, and it remains a growing public health concern both globally and in Taiwan [1]. The prevalence of central obesity rose globally from 31.3% between 1895 and 1990 to 48.3% between 2010 and 2014 and from 27% between 1993 and 1996 to 47% between 2013 and 2016 in Taiwan [1, 2]. It has been strongly associated with metabolic syndrome (MetS), cardiovascular disease, diabetes mellitus (DM), and certain types of cancer, underscoring the importance of identifying related risk factors [1, 3, 4].

The ambient temperature is increasing as a consequence of global warming, with an unprecedented increase over the last 5 decades [5]. High temperatures have been shown to increase the risks of heat exhaustion [6], heatstroke [7], dehydration [8], and cardiovascular disease [9]. High temperatures can also worsen respiratory conditions such as asthma and affect metabolic health, contributing to obesity and diabetes [10, 11]. Moreover, prolonged exposure can lead to chronic kidney damage and mental health issues [12, 13]. In addition to high temperatures, cold temperatures have also been associated with various health problems [14], such as hypothermia [15], cardiovascular disease [15, 16], acute coronary syndrome [17, 18], and respiratory disease [19]. The US Armed Services first introduced the concept of the wet‐bulb globe temperature (WBGT) in the 1950s as a means to address the serious health issues related to exposure to high temperatures [20]. Since its introduction, WBGT has become the most widely used and ISO‐approved heat stress index [21]. WBGT is calculated using natural wet‐bulb temperature, globe temperature, and dry bulb/ambient temperature measurements, thereby taking into account transfer phenomena including evaporation, convection, and radiation [21]. In addition, the WBGT has been shown to be a feasible health evidence–based approach to determine appropriate heat‐warning thresholds with various heat indicators and health outcomes in Taiwan [22, 23]. However, limited research exists on the relationship between WBGT and obesity, particularly in subtropical and tropical regions such as Taiwan. Given the geographical diversity of Taiwan, the WBGT varies significantly between northern, central, southern, and eastern regions due to climatic and topographic factors, consequently understanding the influence of WBGT on central obesity is essential.

This study used data from the Taiwan Biobank (TWB), an extensive population‐based database including more than 120,000 enrollees. There were two main aims of the study: (1) investigate associations between WBGT with central obesity and (2) how these associations differed between different areas of Taiwan. This research provides novel insights into the intersection of environmental health and metabolic disorders, with implications for public health interventions and climate‐adaptive strategies.

## 2. Materials and Methods

### 2.1. Date Source

The Taiwanese Ministry of Health and Welfare devised the TWB to improve healthcare delivery, reduce the burden of chronic diseases, and address issues facing an aging population. The TWB maintains an extensive database containing medical, genetic, and lifestyle data collected from people 30–70 years of age who have no cancer history and reside in diverse communities throughout Taiwan [24, 25]. The project’s ethical standards and procedures were approved by two oversight bodies: the TWB Ethics and Governance Council and Academia Sinica’s Institutional Review Board (IRB) on Biomedical Science Research.

After the individuals consent to participate in the TWB by signing informed consent forms, they undergo a standardized data collection process involving face‐to‐face interviews, physical assessments, and blood sampling. During enrollment, data are recorded including body height (BH), body weight (BW), waist circumference (WC), hip circumference (HC), along with demographics such as age and sex, lifestyle factors such as smoking and drinking habits, existing medical conditions (including hypertension and DM), and educational background. Estimated glomerular filtration rate (eGFR) is calculated with the CKD‐EPI creatinine (Equation (26)), and laboratory tests include fasting glucose, hemoglobin, triglycerides, total cholesterol, low/high‐density lipoprotein (LDL/HDL) cholesterol, and uric acid.

Three measurements of systolic/diastolic blood pressure (SBP/DBP) are also recorded after a 1‐2‐min interval by staff members, with average values being used for analysis. The participants are asked to refrain from ingesting caffeine, smoking, or participating in physical activities for at least 30 min before the BP readings. The study was conducted in accordance with the Helsinki Declaration and approved by the Kaohsiung Medical University Hospital IRB (KMUHIRB‐E (I)‐20240,338).

### 2.2. Study Cohort

This study only included participants who lived on the main island of Taiwan (*n* = 120,424), as those living on offshore islands (*n* = 940) did not have available data on WBGT. The main island of Taiwan is classified into northern (7 cities/counties), central (4 cities/counties), southern (5 cities/counties), and eastern regions (5 counties) [12]. While these areas serve as administrative divisions, climatic differences also exist between them. The southern region is classified as having a tropical climate, whereas the northern and central regions have a subtropical climate. The eastern region is relatively long and narrow and encompasses both subtropical and tropical regions.

### 2.3. WBGT Measurements

There are 453 weather stations across Taiwan, and the Central Weather Bureau provides data from these stations for research purposes [27]. This study used data collected between 2000 and 2020, and WBGT was calculated as WBGT = 0.7*t*_*n* *w*_ +  0.2*t*_*g*_ + 0.1*t*_*a*_, where *t*_*a*_ = dry‐bulb temperature, *t*_*g*_ = globe temperature, and *t*_nw_ = natural wet‐bulb temperature. WBGT values were analyzed during two time periods: work (8:00 a.m.–5:00 p.m.) and midday (11:00 a.m.–2:00 p.m.). A land‐use‐based spatial machine learning model was developed with hourly WBGT measurements as the dependent variable to estimate detailed spatial‐temporal WBGT patterns across Taiwan. Factors possibly influencing WBGT including solar declination, rainfall, relative humidity, and wind speed were also obtained. Land‐use data covering residential, industrial, water and park areas, road networks, and landmarks including restaurants and places of worship were recorded. SHapley Additive exPlanation was used to identify key predictive factors. A prediction model was then constructed combining the identified predictive factors with a light gradient boosting machine. By integrating temperature and land use/cover predictors, the land‐use–based spatial machine learning model achieved exceptional accuracy with an *R* ^2^ value of 0.99 [28] and the ability to generate high‐resolution spatial‐temporal WBGT measurements at 50 × 50 m.

### 2.4. Connecting WBGT Data to Participants in the TWB

We linked TWB participant data with WBGT measurements to assess heat exposure by matching the township of their residential addresses. We calculated average yearly WBGT exposure for each participant, specifically focusing on WBGT values during the year before their enrollment into the TWB. The WBGT values were then used to assess environmental heat exposure.

### 2.5. Definition of Central Obesity

Central obesity was defined as WC ≥ 80 cm for women and ≥ 90 cm for men [29].

### 2.6. Statistical Analysis

Data are given as the percentage or mean ± standard deviation. Categorical and continuous variables were compared with chi‐square and independent *t* tests, respectively. Comparisons amongst regions were analyzed with one‐way analysis of variance followed by Bonferroni’s post hoc test. Uni‐ and multivariable logistic and linear regression analyses were conducted to explore associations between WBGT with central obesity. Statistical analysis was performed using SPSS v25 (IBM Inc., Armonk, NY), with *p* < 0.05 considered significant.

## 3. Results

The mean age of the study cohort (43,247 men and 77,177 women) was 49.9 ± 11.0 years, including 55,927 (46.4%) participants with and 64,497 (53.6%) without central obesity.

### 3.1. Clinical Characteristics of the Study Cohort by Central Obesity

The central obesity group were predominantly male, older, and had higher rates of DM, hypertension, smoking/alcohol habits, BH, BW, WC, HC, SBP and DBP, fasting glucose, hemoglobin, total and LDL‐cholesterol, triglycerides and uric acid, and lower HDL‐cholesterol, educational status, and eGFR than those without central obesity (Table 1). The central obesity group also had higher WBGT during both midday and work periods.

**TABLE 1:** Comparison of clinical characteristics among participants according to central obesity in study participants.

Characteristics	Central obesity (−) (*n* = 64,497)	Central obesity (+) (*n* = 55,927)	*p*
Age (year)	48.8 ± 11.0	51.2 ± 10.8	< 0.001
Male sex (%)	40.4	30.8	< 0.001
DM (%)	5.3	14.3	< 0.001
Hypertension (%)	17.0	33.1	< 0.001
Smoking history (%)	28.0	26.4	< 0.001
Alcohol history (%)	8.3	8.7	0.007
Systolic BP (mmHg)	116.9 ± 17.9	124.5 ± 18.7	< 0.001
Diastolic BP (mmHg)	71.8 ± 11.0	76.1 ± 11.5	< 0.001
BH (cm)	162.1 ± 8.1	161.7 ± 8.5	< 0.001
BW (kg)	58.3 ± 9.5	70.0 ± 13.1	< 0.001
WC (cm)	76.7 ± 6.7	90.9 ± 8.2	< 0.001
HC (cm)	92.3 ± 4.8	100.3 ± 6.9	< 0.001
Education status			< 0.001
Elementary and below (%)	3.4	7.5	
Middle and high school (%)	33.7	40.3	
Collage and above (%)	62.9	52.3	
Laboratory parameters			
Fasting glucose (mg/dL)	93.0 ± 16.1	99.2 ± 24.6	< 0.001
Hemoglobin (g/dL)	13.7 ± 1.6	13.8 ± 1.6	< 0.001
Triglyceride (mg/dL)	98.1 ± 78.0	135.8 ± 106.2	< 0.001
Total cholesterol (mg/dL)	193.0 ± 35.0	198.7 ± 36.6	< 0.001
HDL‐cholesterol (mg/dL)	57.4 ± 13.8	51.3 ± 12.2	< 0.001
LDL‐cholesterol (mg/dL)	117.5 ± 31.0	125.0 ± 32.1	< 0.001
eGFR (mL/min/1.73 m^2^)	106.2 ± 13.5	104.2 ± 14.2	< 0.001
Uric acid (mg/dL)	5.2 ± 1.4	5.7 ± 1.4	< 0.001
Residence			< 0.001
Northern Taiwan (%)	33.6	33.1	
Central Taiwan (%)	19.4	19.0	
Southern Taiwan (%)	36.9	39.1	
Eastern Taiwan (%)	10.1	8.9	
WBGT during noon period (°C)	27.24 ± 1.08	27.27 ± 1.08	< 0.001
WBGT during work period (°C)	25.01 ± 1.17	25.03 ± 1.17	0.011

*Note:* Central obesity was defined as WC > 80 cm in women and > 90 cm in men. Noon period is defined as 11 a.m.–2 p.m., and work period is defined as 8 a.m.–5 p.m. Average WBGT values were recorded for each participant at 1 year before the TWB survey year.

Abbreviations: BH, body height; BP, blood pressure; BW, body weight; DM, diabetes mellitus; eGFR, estimated glomerular filtration rate; HC, hip circumference; HDL, high‐density lipoprotein; LDL, low‐density lipoprotein; WC, waist circumference; WBGT, wet‐bulb globe temperature.

### 3.2. Clinical Characteristics of the Study Cohort by Region

The individuals residing in southern Taiwan had the highest prevalence of central obesity and highest average WBGT values during both midday and work periods compared to the other three regions (Table 2).

**TABLE 2:** Comparison of clinical characteristics among participants in different geographic regions.

Characteristics	Northern Taiwan (*n* = 40,170)	Central Taiwan (*n* = 23,124)	Southern Taiwan (*n* = 45,643)	Eastern Taiwan (*n* = 11,487)	ANOVA *p*
WC (cm)	83.25 ± 10.24	83.29 ± 10.23	83.50 ± 10.19^∗^	82.59 ± 10.39^∗†#^	< 0.001
Central obesity (%)	46.0	45.9	47.9^∗†^	43.2^∗†#^	< 0.001
WBGT during noon period (°C)	2 6.38 ± 0.77	27.40 ± 0.82^∗^	28.10 ± 0.69^∗†^	26.68 ± 1.02^∗†#^	< 0.001
WBGT during work period (°C)	24.83 ± 1.06	23.83 ± 0.74^∗^	25.82 ± 0.78^∗†^	24.87 ± 1.14^∗†#^	< 0.001

*Note:* Central obesity was defined as WC > 80 cm in women and > 90 cm in men. Noon period is defined as 11 a.m.–2 p.m., and work period is defined as 8 a.m.–5 p.m. Average WBGT values were recorded for each participant at 1 year before the TWB survey year.

Abbreviations: WBGT, wet‐bulb globe temperature; WC, waist circumference.

^∗^ *p* < 0.05 compared with northern Taiwan.

^†^ *p* < 0.05 compared with central Taiwan.

^#^ *p* < 0.05 compared with southern Taiwan.

### 3.3. Association Between WBGT With Central Obesity in Each Region

After adjusting for the significant variables shown in Table 1, multivariable logistic regression analysis was performed to examine the associations between WBGT and central obesity in the four geographical regions (Table 3). The results showed that in central Taiwan, each 1°C increase in average midday period WBGT was significantly associated with an increased prevalence of central obesity (odds ratio [OR]: 1.048, *p* = 0.009), whereas each 1°C rise in both midday and work period WBGT in southern Taiwan was significantly associated with a low prevalence of central obesity (OR: 0.929, *p* < 0.001 and 0.954, *p* < 0.001, respectively). However, no significant associations were observed between central obesity with WBGT in northern or eastern Taiwan. The associations between WBGT and central obesity during midday and work periods by region are shown in Figures 1(a) and 1(b), respectively.

**TABLE 3:** Association of WBGT and central obesity in different geographic regions using multivariable logistic regression analysis.

WBGT	Central obesity odds ratio (95% confidence interval)	*p*
	Northern Taiwan	
WBGT during noon period (per 1°C)	0.995 (0.967–1.023)	0.713
WBGT during work period (per 1°C)	1.015 (0.994–1.036)	0.164
	Central Taiwan	
WBGT during noon period (per 1°C)	1.048 (1.012–1.087)	0.009
WBGT during work period (per 1°C)	1.033 (0.933–1.074)	0.110
	Southern Taiwan	
WBGT during noon period (per 1°C)	0.929 (0.901–0.958)	< 0.001
WBGT during work period (per 1°C)	0.954 (0.928–0.980)	0.001
	Eastern Taiwan	
WBGT during noon period (per 1°C)	1.018 (0.977–1.060)	0.393
WBGT during work period (per 1°C)	0.991 (0.955–1.027)	0.609

*Note:* Values expressed as odds ratio and 95% confidence interval. Abbreviations: WBGT, wet‐bulb globe temperature. Central obesity was defined as WC > 80 cm in women and > 90 cm in men. Noon period is defined as 11 a.m.–2 p.m., and work period is defined as 8 a.m.–5 p.m. Average WBGT values were recorded for each participant at 1 year before the TWB survey year. Multivariable model: adjusted for age, sex, smoking and alcohol history, diabetes, hypertension, systolic and diastolic BP, education status, fasting glucose, hemoglobin, triglycerides, total cholesterol, HDL‐cholesterol, LDL‐cholesterol, eGFR, and uric acid (significant variable in Table 1).

FIGURE 1(a) Forest plots showing the associations between WBGT and central obesity by geographic region during the midday period in multivariable linear regression analysis. (b) Forest plots showing the associations between WBGT and central obesity by geographic region during the work period in multivariable linear regression analysis.

**Figure:**
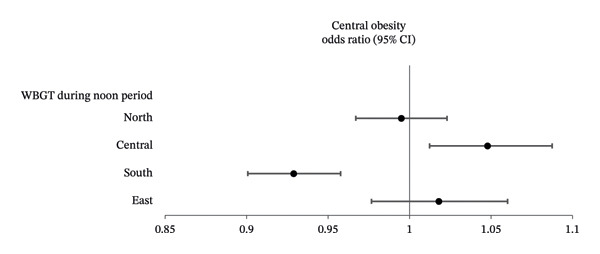
(a)

**Figure:**
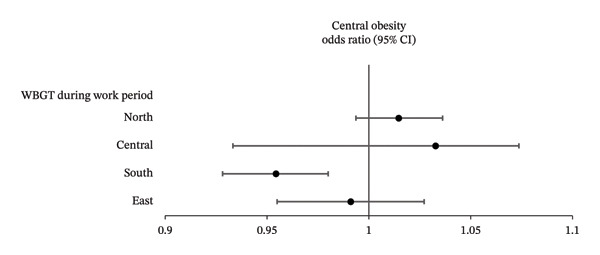
(b)

We further performed the association of relative humidity (%) and central obesity in different geographic regions using multivariable logistic regression analysis. The results showed that in central Taiwan, each 1% increase in relative humidity was significantly associated with an increased prevalence of central obesity (OR: 1.022, *p* < 0.001 in northern Taiwan; OR: 1.029, *p* < 0.001 in central Taiwan; and OR: 1.052, *p* < 0.001 in southern Taiwan), whereas each 1% increase in relative humidity was significantly associated with a low prevalence of central obesity (OR: 0.913, *p* = 0.002 in eastern Taiwan).

## 4. Discussion

This study examined associations between WBGT and central obesity in four regions of Taiwan, and the results showed significant regional differences. High WBGT was associated with a high prevalence of central obesity in central Taiwan but a low prevalence in southern Taiwan, and no significant associations were found in northern or eastern Taiwan.

The findings of the associations between high WBGT and a high prevalence of central obesity in central and northern regions align with prior studies suggesting that climatic factors, and particularly heat, can influence the prevalence of obesity [11]. Previous research has indicated that high temperatures may promote central obesity through metabolic and behavioral mechanisms. Yang et al. explored the link between obesity and ambient temperature in 124,354 participants in Korea [30]. Overall obesity was defined as a BMI ≥ a025 kg/m^2^, and abdominal obesity as a WC ≥ 90/85 cm for men/women, and their results showed positive correlations between BMI, WC, and mean annual temperature (MAT). Participants in the highest MAT quintile (14.1°C–16.6°C) had a 1.045 times higher odds of overall obesity and a 1.082 times higher odds of abdominal obesity compared to lower MAT groups. The authors concluded that higher temperatures may reduce the energy expenditure required for thermogenesis, potentially contributing to increased obesity [30]. In addition, heat exposure has been associated with reduced physical activity levels, leading to increased abdominal fat deposition [31]. Kim et al. reported that a 1°C increase in daily maximum temperature reduced the amount that people walked in both rural (OR 0.95) and urban (OR 0.98) areas [31]. Furthermore, high temperatures appear to impact the regulation of hormones involved in energy balance and obesity, such as thyroid‐stimulating hormone (TSH). TSH significantly stimulates the secretion of leptin, a hormone produced by human adipose tissue that suppresses appetite and reduces body weight [32]. In vitro studies have demonstrated this leptin‐stimulating effect of TSH at concentrations of 10^−9^ M (*p* < 0.0005), 10^−11^ M (*p* < 0.05), and 10^−13^ M (*p* < 0.005) after 48 h of incubation [33]. However, another study demonstrated a reduction in TSH level by 3.7% (95% CI: −6.4%, −0.9%) at 29.8°C compared with the reference temperature (13.3°C) [34]. This decrease in TSH under high temperatures may weaken its ability to stimulate leptin secretion, potentially disrupting the hormonal pathways that regulate appetite, body weight, and energy metabolism, thereby increasing obesity. In central and northern Taiwan, elevated WBGT may promote a sedentary lifestyle, particularly in urbanized areas where air conditioning is prevalent. While indoor temperatures may be regulated, reduced physical activity in climate‐controlled environments could increase the risk of central obesity [35, 36]. Lastly, compared with southern Taiwan, the colder winter temperatures in central and northern Taiwan may encourage residents to prefer high‐calorie diets for warmth, leading to excessive energy intake and an increased risk of obesity [37].

However, in tropical climates, prolonged exposure to high temperatures may trigger adaptive thermogenic responses, resulting in higher basal metabolic rates and reduced fat accumulation [38, 39]. The second finding of this study is that high WBGT was associated with a low prevalence of central obesity in southern Taiwan. Compared with central, northern, and eastern regions, the WBGT was highest in southern Taiwan during both midday and work periods. The findings in southern Taiwan support the hypothesis of an adaptive thermogenic response, as the consistently high WBGTs in this tropical region likely facilitate energy expenditure through heat adaptation [40]. In addition, an elevated temperature can decrease appetite, potentially contributing to a reduction in central obesity. A previous study showed that broiler chicks exposed to high ambient temperatures consumed significantly less food (approximately 50%) compared with those in thermoneutral conditions (*p* < 0.05) at both 30 and 90 min of exposure [41]. Lastly, Xiaofang et al. reported that chronic heat exposure significantly altered intestinal morphology, characterized by a reduction in villus height and increase in crypt depth in the small intestine, resulting in a lower villus height/crypt depth ratio. This change was found to compromise nutrient absorption by reducing the surface area available for uptake, with the impaired absorptive capacity leading to malabsorption and subsequent weight loss. In heat‐stressed broiler chicks, these morphological changes were linked to reduced average daily feed intake and growth performance, highlighting the critical role of intestinal damage in growth limitations under heat stress [42]. Moreover, there are high rates of industrial development and manual workers in southern Taiwan, leading to a general increase in physical activity and consequently a lower prevalence of central obesity [43, 44]. Finally, the warm and humid environment in southern Taiwan is more suitable for the spread of infectious diseases such as dengue fever and enterovirus [45]. These infections may lead to weight loss or influence eating behaviors, potentially reducing the prevalence of obesity. However, we tried to analysis the association between relative humidity and central obesity, and the results showed that increase in relative humidity was significantly associated with an increased prevalence of central obesity in southern Taiwan, whereas each 1% increase in relative humidity was significantly associated with a low prevalence of central obesity (OR: 0.913, *p* = 0.002) in eastern Taiwan. This finding contradicted our hypothesis. Further longitudinal studies and more precise analysis are needed, including usual dietary habits; then, we can better explain the cause‐and‐effect relationship.

Our findings appear to be contradictory in that in central Taiwan, each degree increase in WBGT contributed to central obesity, whereas in the even hotter southern regions, each degree increase in WBGT reduced the incidence of central obesity. However, this can be explained by studies examining the relationship between temperature and metabolic rate. Douglas et al. demonstrated that metabolism increases when temperatures rise above or fall below the thermoneutral zone, a range of comfortable temperatures that minimizes the need for active thermoregulation and energy expenditure [46]. Another relevant study by Zhen et al. highlighted that temperature was associated with cardiovascular diseases and often followed a “J,” “U,” or “V” shaped relationship [47]. These findings may explain the seemingly paradoxical effect of WBGT on central obesity: in central and northern Taiwan, WBGTs are closer to the thermoneutral zone, minimizing energy expenditure and promoting fat storage. In contrast, the higher WBGTs in southern Taiwan exceed the thermoneutral zone, increasing energy expenditure and thereby reducing the risk of central obesity.

The main strengths of this study include the large number of people residing across the four main regions of Taiwan and comprehensive data on weather conditions to calculate the WBGT. There were also several limitations. First, the cross‐sectional nature of the study precluded further analysis of temporal relationships between WBGT with central obesity. Further longitudinal investigations are needed to confirm our findings. Second, data on prescriptions of medications that could influence hypertension, fasting glucose, lipid profiles, and obesity are not recorded in the TWB; this may have affected our findings. Third, we were only able to estimate outdoor but not indoor WBGT, which could have affected assessments of WBGT exposure due to the use of dehumidifiers and air conditioning indoors. However, a previous study reported a strong correlation between indoor and outdoor temperature when the outdoor temperature exceeds 12.7°C, so we believe this would not have affected our results [48]. Fourth, we lack the information of occupation type (outdoor manual/indoor manual/service/office), income, and urban/rural status, which could provide more information to strengthen our findings and help us to distinguish between direct physiological WBGT effects and behavioral/occupational pathways. Finally, as women tend to be more able or willing to participate in research studies such as the TWB compared with men, our findings may not be generalizable to the general population.

In conclusion, our results highlight the complex interplay between climatic factors and metabolic health. The regional differences in the relationship between WBGT and central obesity underscore the value of tailored public health strategies to address climate‐related health risks. Longitudinal studies are needed to confirm our findings and inform adaptive interventions for obesity prevention across diverse climates and regions.

## Author Contributions

Conceptualization, methodology, validation, formal analysis, writing–review and editing, and supervision: Y‐W.C., J‐M.C., C‐D.W., and S‐C.C. Software and investigation: S‐C.C. Resources, project administration, and funding acquisition: S‐C.C. Data curation: S‐H.C., W‐Y.S., P‐H.W., C‐Y.H., M‐Y.L., Y‐W.C., J‐M.C., C‐D.W., and S‐C.C. Writing–original draft preparation: S‐H.C. and S‐C.C. Visualization: C‐D.W. and S‐C.C.

## Funding

This work was supported partially by the Research Center for Precision Environmental Medicine, Kaohsiung Medical University, Kaohsiung, Taiwan, from the Featured Areas Research Center Program within the framework of the Higher Education Sprout Project by the Ministry of Education in Taiwan and by Kaohsiung Medical University Research Center Grant (KMU‐TC14A01) and Kaohsiung Municipal Siaogang Hospital (S‐113–06).

## Disclosure

All authors have read and agreed to the published version of the manuscript.

Part of this work was previously presented as a conference abstract at the Annual Meeting of the Taiwan Society of Internal Medicine [49].

## Ethics Statement

The study was conducted according to the Declaration of Helsinki, and it was granted approval by the Institutional Review Board of Kaohsiung Medical University Hospital (KMUHIRB‐E (I)‐20240,338), and the TWB was granted approval by the IRB on Biomedical Science Research, Academia Sinica, Taiwan, and the Ethics and Governance Council of the TWB.

## Consent

Informed consent to participate was obtained from all of the participants in the study.

## Conflicts of Interest

The authors declare no conflicts of interests.

## Data Availability

The data underlying this study are from the Taiwan Biobank. Due to restrictions placed on the data by the Personal Information Protection Act of Taiwan, the minimal dataset cannot be made publicly available. Data may be available upon request to interested researchers. Please send data requests to Szu‐Chia Chen, PhD, MD, Division of Nephrology, Department of Internal Medicine, Kaohsiung Medical University Hospital, Kaohsiung Medical University.
